# Slope-Based Stochastic Resonance: How Noise Enables Phasic Neurons to Encode Slow Signals

**DOI:** 10.1371/journal.pcbi.1000825

**Published:** 2010-06-24

**Authors:** Yan Gai, Brent Doiron, John Rinzel

**Affiliations:** 1 Center for Neural Science, New York University, New York, New York, United States of America; 2 Department of Mathematics, University of Pittsburgh, Pittsburgh, Pennsylvania, United States of America; 3 Center for the Neural Basis of Cognition, University of Pittsburgh, Pittsburgh, Pennsylvania, United States of America; 4 Courant Institute of Mathematical Science, New York University, New York, New York, United States of America; Université Paris Descartes, Centre National de la Recherche Scientifique, France

## Abstract

Fundamental properties of phasic firing neurons are usually characterized in a noise-free condition. In the absence of noise, phasic neurons exhibit Class 3 excitability, which is a lack of repetitive firing to steady current injections. For time-varying inputs, phasic neurons are band-pass filters or slope detectors, because they do not respond to inputs containing exclusively low frequencies or shallow slopes. However, we show that in noisy conditions, response properties of phasic neuron models are distinctly altered. Noise enables a phasic model to encode low-frequency inputs that are outside of the response range of the associated deterministic model. Interestingly, this seemingly stochastic-resonance (SR) like effect differs significantly from the classical SR behavior of spiking systems in both the signal-to-noise ratio and the temporal response pattern. Instead of being most sensitive to the peak of a subthreshold signal, as is typical in a classical SR system, phasic models are most sensitive to the signal's rising and falling phases where the slopes are steep. This finding is consistent with the fact that there is not an absolute input threshold in terms of amplitude; rather, a response threshold is more properly defined as a stimulus slope/frequency. We call the encoding of low-frequency signals with noise by phasic models a slope-based SR, because noise can lower or diminish the slope threshold for ramp stimuli. We demonstrate here similar behaviors in three mechanistic models with Class 3 excitability in the presence of slow-varying noise and we suggest that the slope-based SR is a fundamental behavior associated with general phasic properties rather than with a particular biological mechanism.

## Author Summary

Principal brain cells, called neurons, show a tremendous amount of diversity in their responses to driving stimuli. A widely present but understudied class of neurons prefers to respond to high-frequency inputs and neglect slow variations; these cells are called phasic neurons. Although phasic neurons do not normally respond to slow signals, we show that noise, a ubiquitous neural input, can enable them to respond to distinct features of slow signals. We emphasize the fact that, in the presence of noise, they are still sensitive to the change in stimulus, rather than to the constant part of the slow inputs, just as they are for fast inputs without noise. This feature distinguishes the response of phasic neurons from those of other neurons, which show more sensitivity to the amplitude of their inputs. We believe that our study has significantly broadened the understanding about the information-processing ability and functional roles of phasic neurons.

## Introduction

Stochastic resonance (SR) has been extensively described in both bi-stable and excitable systems and is a classic example of noise enhanced processing [1]–[9]. Briefly, SR involves noise facilitating dynamic state transitions or threshold crossing, while permitting phase-locked response to a subthreshold signal. The interaction of signal, noise, and response nonlinearity maximizes signal encoding at a nonzero value of noise intensity. Here, we characterize the novel manner in which SR-like phenomena occur in phasic neuron models. Phasic neurons are characterized by the absence of repetitive firing to steady current injection and low-frequency input, yet show faithful responses to brief pulsatile and high-frequency signals [10]–[13]. In a classical SR system, often exemplified by non-phasic neurons, a signal can be detected without noise simply by making its amplitude adequately large. In contrast, deterministic phasic neurons will not respond to low-frequency input even if the signal amplitude is very large, making phasic neurons an ideal framework to study noise-gated coding [14]. We convey our insights by presenting detailed results for a phasic model [15] that has been widely used in modeling various auditory brainstem phasic neurons [16]–[18] that perform precise temporal processing and respond only to rapid transients and coincidences. We then examine other types of phasic models, showing that our findings are general.

The Class 3 excitability, which is commonly used to define phasic responses [12], can be created by different cellular mechanisms, such as recruiting a low-threshold potassium current (*I*_KLT_) [19]–[22], inactivating the sodium current (*I*_Na_) [12], [23]–[24], or steepening the activation of the high-threshold fast potassium current (*I*_KHT_) [25]. The phasic neuron model that is our primary focus here is a Hodgkin-Huxley (HH) type model with *I*_KLT_ [15]. Combining the same phasic neuron model and whole-cell recordings in the medial superior olive (MSO) in gerbil, we have previously shown that adding noise enables phasic neurons to detect low-frequency inputs, which, alone, cause no spiking response [14]. Although this behavior seems to be consistent with SR, it is fundamentally different from SR for the reasons listed below.

In a classical SR system, adding a small amount of noise to a subthreshold signal facilitates threshold crossing, such as a spike emission upon crossing a membrane voltage (*V*_m_) threshold. When the intensity of the noise is properly chosen, the signal can be encoded by eliciting more spikes around the signal's peak and fewer spikes around its trough. The larger the amplitude of the subthreshold signal, the better the noise-gated encoding becomes; whereas, for supra-threshold signals noise will only degrade signal encoding. In this sense, we call the classical SR system an “amplitude-based stochastic resonance”. However, we discovered that phasic MSO neurons and a phasic neuron model [15] responded to the rising, falling, or trough phases, depending on the spectrum of the noise, but not to the signal's peak except for very large noise [14]. Here, we report that an essential feature of phasic neurons is that response “threshold” is better defined in terms of the slope rather than the amplitude of the input. We further show that the noise-gated signal encoding is sensitive to the slope of the signal, as opposed to its amplitude. For this reason, we label SR-like phenomena in phasic neurons as a “slope-based stochastic resonance”.

In this study, we highlight the novelty of a phasic neuron's slope-based SR behavior by contrasting it with the qualitatively distinct amplitude-based SR and coding properties of tonic neurons. To this end, we first compare the dependence of the signal-to-noise ratio (SNR) on noise intensity obtained from a tonic model to that from a phasic model. In addition to analyzing SNRs, we pay attention to the temporal firing patterns, which are often overlooked when SR systems are concerned. Next, we show that the slope-based SR behavior of the phasic model can be reflected in a highly non-monotonic *f*-*I* (firing rate vs. stimulus mean) relation with the compelling feature that firing rate falls continuously to zero with increasing *I*. Such *f-I* curves have been reported for phasic neurons and models [24], [26], [27]. Finally, the slope threshold in response to a ramp stimulus, as is observed in noise-free conditions [28], is reduced or diminished by the addition of noise. In total, we report that the influence of noise and any noise-assisted coding performed by phasic neurons is significantly distinct from that of tonic neurons.

The occurrence of Class 3 excitability is often associated with outward currents, i.e., *I*_KLT_ or *I*_KHT_ [25], [29]. It is far less realized that strong inactivation of *I*_Na_ can also create Class 3 excitability [23]. We show that the slope-based SR behavior is observed with phasic models created by manipulating the voltage dependency of either *I*_KHT_ or *I*_Na_ when the noise spectrum favors low frequencies. Our present study, complemented by our previous experimental results [14], reveals that phasic neurons can have substantially different behaviors in noisy conditions compared to their behaviors in non-noisy conditions. The conventional views of phasic neurons being band-pass filters or slope detectors, which are all acquired in idealized conditions with no noise present, should be re-evaluated in noisy conditions.

## Results

### Tonic and Phasic Models Behave Differently in a Noise-Free Condition

**Figure 1:**
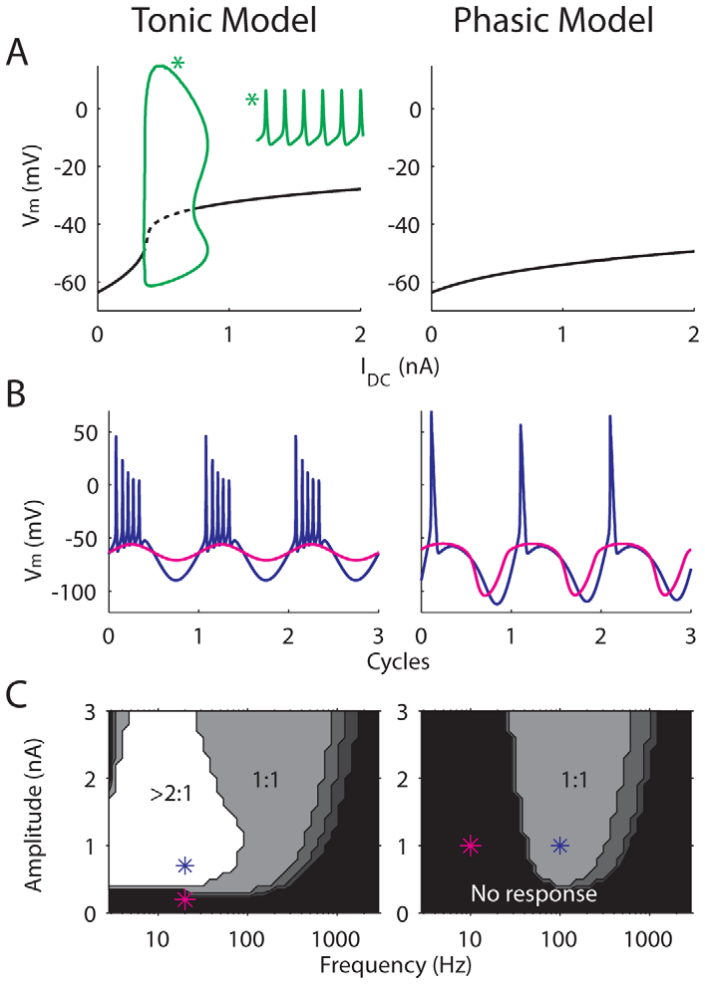
Basic property of the tonic and the phasic models in response to simple current injections. (A) Bifurcation diagrams of the tonic (*left*) and phasic (*right*) models obtained with steady-state current (*I*_DC_). Solid lines represent stable equilibrium. The tonic model displays repetitive firing over a range of *I*_DC_ (green). The inset marked with * is a 20-ms voltage trace obtained when *I*_DC_ = 0.6 nA. (B) and (C) Responses of the tonic (*left*) and phasic (*right*) models to sinusoidal inputs with zero mean replotted from [14]. (B), two voltage traces over three stimulus cycles for input amplitude and frequency marked in the *lower* panels (*). (C) frequency-response maps of the models. Gray-scale colors represent spiking ratios (number of spikes over number of cycles) to a sinusoid current input with varying frequency (x axis) and amplitude (y axis).

The response or bifurcation diagram of the tonic model shows repetitive firing over a range of steady current input, *I*_DC_ (Fig. 1A, *left*, green). An example time course (*I*_DC_ = 0.6 nA) is plotted as an inset. In contrast, the phasic model shows typical Class 3 excitability (Fig. 1A, *right*) by having a unique stable steady state for all *I*_DC_. Note that the “phasicness” of the phasic model is relatively strong [14] so that no repetitive firing is observed even for large steady input current, unlike in some previous studies [29]–[31].

The phasic and tonic models also show different firing preferences for sinusoidal input with varying frequency and amplitude (Fig. 1B and C; replotted from Fig. 1 in [14]). For the tonic model, the input threshold (the lowest amplitude of a sinusoid that causes firing) remains relatively constant for low frequencies. In contrast, the input threshold of the phasic model rises sharply on the low-frequency side. For this reason, phasic neurons are commonly viewed as band-pass filters, and consequently it is difficult to define a universal input threshold in terms of input amplitude. The threshold rise is not completely amplitude independent because 1) increasing the amplitude of a sinusoid steepens the zero-crossing slope, and 2) increasing the amplitude of a sinusoid is similar to decreasing the pre-ramp holding current of a ramp stimulus, which leads to decreased slope threshold. Nevertheless, for phasic neurons it is more natural to define the threshold in terms of an input slope/frequency.

Another distinction between tonic and phasic firing is the spiking ratio (the number of spikes per stimulus cycle) for low-frequency sinusoidal inputs (Fig. 1C). The tonic model fires more than one spike for low-frequency inputs (*left*), whereas the phasic model fires only one spike in each cycle for most of the input conditions (*right*). Representative time courses are plotted in Fig. 1B. Therefore, even if the phasic model responds to low-frequency inputs with extremely large input amplitude, the firing rate is low (e.g., 20 spikes/sec for a 20-Hz sinusoid with 4-nA peak amplitude). Based on this feature, later we show that the intensity of noise that optimizes signal encoding is different for the tonic and phasic models.

**Figure 2:**
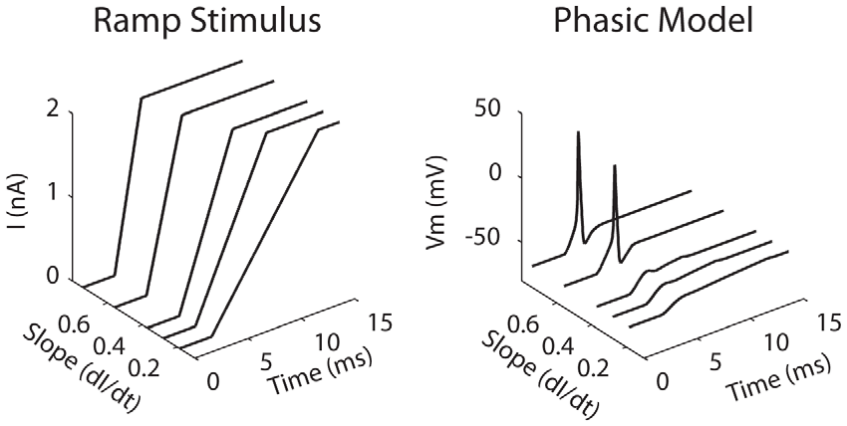
Voltage traces of the phasic model (*right*) in response to ramp stimulus with different slopes (*left*).

Phasic neurons are often called slope detectors because they respond to fast-rising, but not to slow-rising, ramps [28]. Fig. 2 shows the *V*_m_ of the phasic model (*right*) in response to ramp current with different slopes (*left*). The ramp elicits an action potential only when its slope (dI/dt) exceeds 0.55 nA/ms. In contrast, the tonic model fires action potentials to ramps with any slope, as long as the input amplitude reaches 0.3 nA (not shown).

### Noise Can Gate the Encoding of Low-Frequency Signals

**Figure 3:**
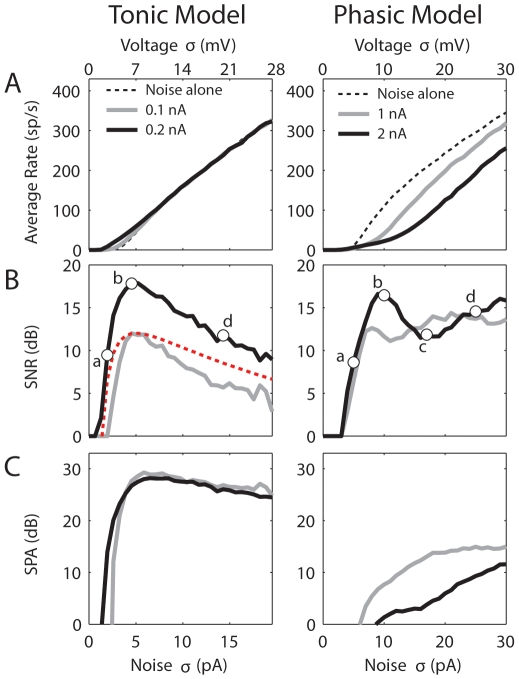
Average firing rate (A), SNR (B), and SPA (C) vs. standard deviations of noise (bottom horizontal axis) and of *V*_m_ (top axis) for the tonic (*left*) and phasic (*right*) models. The red dotted line is a fit to the SNR for the smaller signal amplitude using Equation 4. The voltage σ was obtained separately when the spiking mechanism was disabled by setting the activation/inactivation variables of the *I*_Na_ and *I*_KHT_ to their resting values. The points and letters marked in the lower panels indicate the noise *σ* values (tonic model, *σ* = 2, 5, and 14 pA for ***a***, ***b***, and ***d***; phasic model, *σ* = 5, 10, 17 and 25 pA for ***a***, ***b***, ***c***, and ***d***) that are used in Fig. 4. The legends show signal amplitude. Signal frequency was 20 Hz.

Average firing rate and SNR are presented first to provide a general measure of the model behaviors, followed by detailed frequency and temporal responses. Fig. 3A shows that the average firing rate of both models increased monotonically with noise intensity (*σ*). When the signal amplitude increased from 0.1 to 0.2 nA, the firing rate of the tonic model remained constant except at very low noise intensities. In contrast, when the amplitude of the signal increased from 1 to 2 nA, the firing rate of the phasic model decreased substantially. The relationship between firing rate and signal amplitude will be explored more thoroughly later.

Fig. 3B shows the SNR obtained with the larger (black) and smaller (gray) signal amplitudes for both models. The SNR of the tonic model resembled the SNR of a classical SR system, showing an abrupt rise before a peak and a gradual decay after the peak [4], [32]. In addition, the peaks of the SNRs for both signal amplitudes were obtained at the same noise intensity (*σ* = 5 pA), consistent with an asymptotic theory of SR for weak signals (see Equation 4). The red dotted line is a fit to the SNR for the smaller signal amplitude (black) using Equation 4. Although the SNR decayed faster than the fit, they had essentially the same shape. In contrast, the SNRs obtained with the phasic model did not show classical SR-like behavior (Fig. 3B, *right*). For both signal amplitudes, the SNRs did not decrease significantly at high noise intensities. Presumably, the SNR will drop for very large noise intensity, however the membrane fluctuation caused by the largest noise intensity used here (*σ* = 30 pA) already reached 30 mV for the phasic model (Fig. 3A, *right*, top horizontal axis). For the larger signal amplitude (black), a distinct dip occurred in the SNR around *σ* = 17 pA (marked with ***c***), which yielded a SNR even lower than the SNR obtained with the smaller signal amplitude (gray). Thus it is impossible to fit the SNR of the phasic model using Equation 4. To understand why the SNRs of the phasic model had such unusual shapes, later we show more detailed frequency and temporal response patterns for the larger signal amplitude. Responses at several representative noise intensities (marked in the SNR plots) were chosen for demonstration.

**Figure 4:**
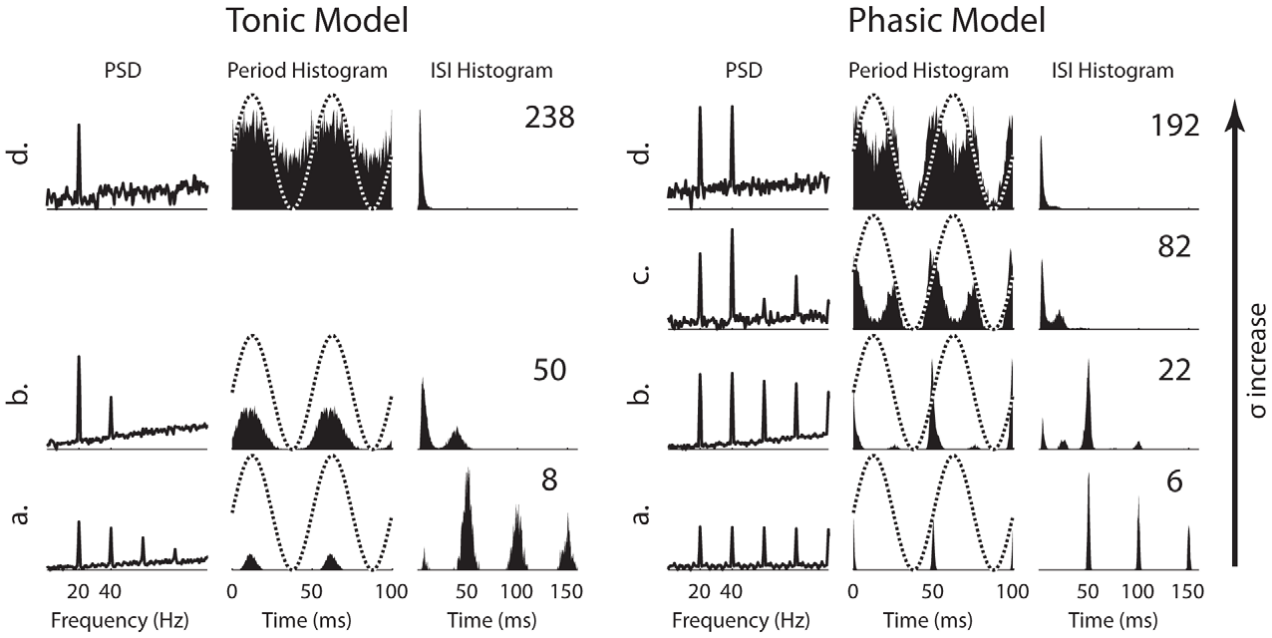
Comparisons of the tonic and phasic models for power-spectrum density (PSD), period histogram, and inter-spike interval (ISI) histogram at different representative noise levels (specified in **Fig. 3**). The amplitude of the signal was 0.2 and 2 nA for the tonic and phasic models, respectively. Signal frequency was 20 Hz. The dotted lines in the period histogram plots represent the time course of the sinusoidal signal for illustration purpose. Two identical cycles of period histograms are plotted. The scale of the vertical axes of the PSD and period histogram plots are fixed over all the panels. The scale of the vertical axes of the ISI histogram plots are not fixed due to a large variation of values across panels. The average firing rate (sp/s) is marked in the upper right corner of each panel.

Here the SNR reflects at the system's output the signal power with respect to the noise power. Another frequently used metric in studies of SR is the spectral power amplification (SPA) [9]: the peak power at the signal's fundamental frequency normalized by the total signal power (see Methods), a measure of gain of the subthreshold signal. For the tonic model, the SPA behaved similar to the SNR in that an optimal value of noise intensity can be identified to yield the highest signal gain (Fig. 3C, *left*). Moreover, the amplification of the signal was approximately constant with respect to signal amplitude, as increasing the signal amplitude from 0.1 (gray) to 0.2 nA (black) did not noticeably change the gain. In contrast, the SPA for the phasic model kept increasing for a fixed signal amplitude up to the highest noise level tested (Fig. 3C, *right*). In other words, with increasing noise intensity, the signal's gain was also increasing, leaving a relatively flat SNR (Fig. 3B, *right*); there was no optimal noise intensity to achieve the highest signal's gain. Another striking feature was that, as the signal amplitude increased from 1 (gray) to 2 nA (black), the SPA as a measure of the signal's gain decreased significantly (Fig. 3C, *right*). As will be described below, this was because for the larger signal amplitude, more output power was shifted from the signal's fundamental frequency to the first harmonic. Finally, it should be noted that because the phasic model did not favor low-frequency signals, the SPA as a measure of the signal's gain was considerably smaller than the SPA for the tonic model (Fig. 3C). For the tonic model, the power-spectrum density (PSD) plots agreed with PSDs from classical SR systems with weak signals [4] in that a large peak occurred at the signal frequency (20 Hz) with smaller peaks at the harmonics for low-intensity noise (Fig. 4, *left*, 1^st^ column). The period histograms showed highest sensitivity to the signal's peak at all noise intensities with more uniformly distributed spikes occurring at high noise intensities (Fig. 4, *left*, 2^nd^ column). The interspike-interval (ISI) histograms indicated that missing signal cycles only occurred on the rising phase of the SNR curve (***a***); once the SNR reached its peak (***b***), there were always one or more spikes in each cycle (***b*** and ***d***) (Fig. 4, *left*, 3^rd^ column). All of these behaviors were consistent with what are expected for neurons exhibiting classical SR behaviors [1].

In contrast, the phasic model was mostly sensitive to the signal's rising phase, indicated by the period histograms (Fig. 4, *right*, 2^nd^ column). After SNR reached its peak (***b***), the phasic model also responded to the signal's falling phase with a lower firing probability compared to the responses in the rising phase (***c*** and ***d***). This preference for two distinct phases explained why the power at the first harmonic can be larger than the power at the fundamental frequency for certain noise intensities (Fig. 4, *right*, 1^st^ column, ***c***). This also explained why there was a peak at half signal cycle in the ISI histogram for certain noise intensities (***b*** and ***c***). In addition, for very high noise intensities (***d***), the phasic model still showed no response in the signal's trough, which is consistent with high SNR persisting at high noise intensities (Fig. 3B, *right*).

Note that these distinct features were observed with the phasic model for low-frequency signals. As the signal frequency increased, the SNR behaved more similar to the SNR of a classical SR system, and responses occurred around only one phase of each signal cycle (e.g., for 100 Hz, not shown). The above descriptions for the tonic model were generally independent of frequency.

**Figure 5:**
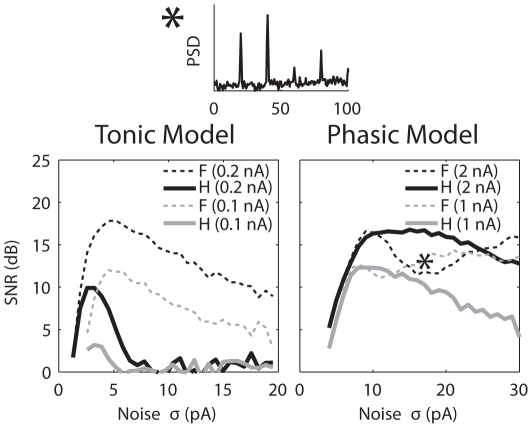
SNR computed at the first harmonic of the signal frequency (thick solid lines in the bottom panels) for condition *c* in **Fig. 4**. The small top panel shows the PSD at the noise level marked with the star in the SNR plot on the right. For comparison, SNR computed at the fundamental of the signal frequency (thin dotted lines) are re-plotted from Fig. 3. *F*, fundamental (20 Hz). *H*, harmonic (40 Hz).

To make a more detailed comparison between the signal coding at the fundamental frequency and the first harmonic, the SNRs computed at the first harmonic are plotted in Fig. 5. For the tonic model (*left*), the SNRs at the first harmonic (solid) were always lower than the SNRs at the fundamental frequency (dotted). This was also the case for the phasic model with the lower signal amplitude, except around the peak of the SNR (Fig. 5, *right*, gray). With the higher signal amplitude, there was a range of noise level (∼15 to 25 pA) that yielded a higher response power value at the first harmonic than at the fundamental frequency (Fig. 5, *right*, black).

In summary, the impact of noise on the encoding of low-frequency signals was different between the phasic and tonic models. In classical SR studies SNR is normally measured at the fundamental frequency of the signal and therefore does not capture how stimuli shape the temporal pattern of responses in other frequency bands. In particular, for large-amplitude stimuli there can be significant stimulus-response interactions at frequencies outside the stimulus spectrum, a hallmark of a nonlinear stimulus-response transfer function. The unusual SNR curves produced by the phasic model (Fig. 3B, *right*) are caused by significant firing at double the signal frequency for some noise intensities. Thus, the dip of the SNR computed from the fundamental frequency (marked with ***c***) did not mean that the signal was badly encoded, but meant that it was encoded at a harmonic frequency of the fundamental. Although in some previous studies [9], , nonlinear SR has been considered and quantified at higher harmonics, those studies did not associate such measurements with a clear temporal pattern, e.g., firing at the rising and/or falling phases, as shown in the present study.

**Figure 6:**
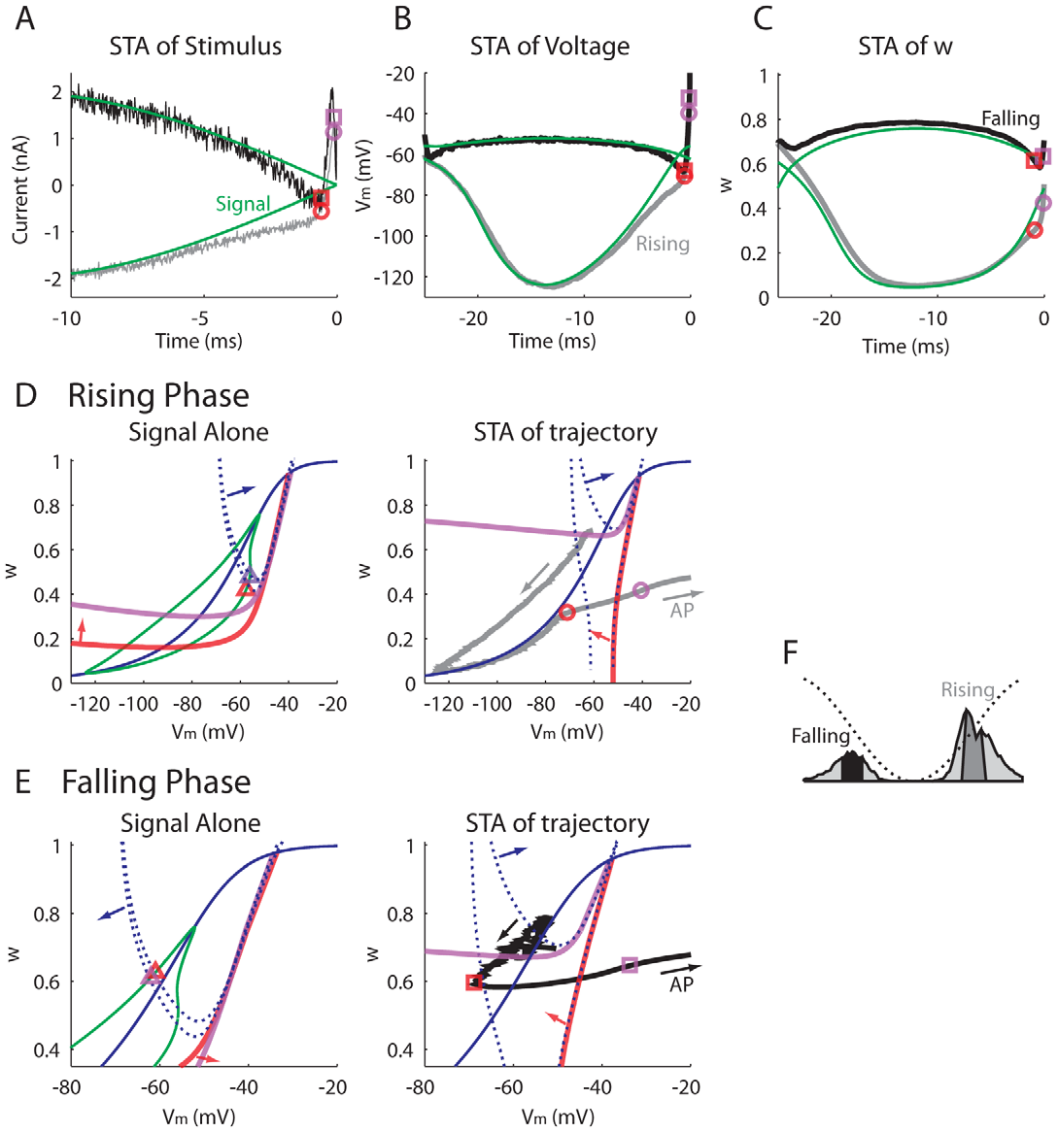
Spike-triggered averages (STAs) for spikes occurring in a 4-ms window centering at the rising (gray) and falling (black) phases of the 20-Hz signal (*A_s_* = 2 nA) for the phasic model. The signal alone and its responses are plotted in green. (A) STA of stimulus. (B) STA of *V*_m_. (C) STA of *w*, which is the fast gating variable of the *I*_KLT_. (D) and (E) Voltage-*w* phase-plane analysis. Two phase points, one before and one after the initiation of the averaged action potential (AP) for the rising or falling phase are marked with circles and squares, respectively. The corresponding phase points in the signal's trajectory are marked with triangles (*I* = −150 and −25 pA for the rising phase; *I* = 150 and 25 pA for the falling phase). Blue dotted, *V*_m_ nullcline. Blue solid, *w* nullcline. Red and purple, threshold separatrix. (F) Period histogram showing the selection of spikes. Note that only spikes with a previous inter-spike interval longer than half of the signal cycle were included to avoid averaging action potentials with subthreshold *V*_m_. The stimulus condition is as marked with ***c*** in Fig. 3. Stimulus duration was 500 s. Noise *σ* was 15 pA.

We gain insight into the phasic model's unusual response properties by applying reverse correlation analysis and examining the spike-triggered averages (STA) of several dynamic quantities: the stimulus (Fig. 6A), *V*_m_ (Fig. 6B), the fast gating variable, *w*, of *I*_KLT_ (Fig. 6C), and the system's trajectory in the *V*_m_-*w* phase plane (Fig. 6D and E) for condition ***c*** (Fig. 4). We select from a brief time window (4 ms) centered on the rising or falling phases of the signal (Fig. 6F).

The stimulus STA indicates that, on average, a modest hyperpolarizing dip preceded the strong brief depolarizing component just prior to spike initiation (Fig. 6A), consistent with previous findings [14], [22], [23]. As seen in the period histograms above (Fig. 4, *right*, 2^nd^ column), the phasic model barely responded to the signal's peak for a wide range of noise intensity. This lack of response was due to the activation of *I*_KLT_, indicated by the high values of *w* (Fig. 6C, black) and the nearly flat voltage traces (Fig. 6B, black) during the positive half of the sinusoid. For spikes occurring on the signal's rising phase, the rises in *V*_m_ and *w* just before spike initiation were significantly slowed by the noise (gray) in comparison to the *V*_m_ and *w* responses to signal without noise (green). For spikes occurring on the signal's falling phase, the hyperpolarizing noise dip led, on average, to a faster decrease in *w* before a spike (black) compared to the decrease of *w* caused solely by the signal (green) (Fig. 6C).

These observations can be rationalized by phase plane analysis, by comparing features and trajectories in the STA of *V*_m_-*w* phase plane (Fig. 6D and E, *right*), with those of the deterministic phase trajectory of the signal-induced (noise-free) response (Fig. 6D and E, *left*). Due to the presence of *I*_KLT_, there is not a fixed voltage threshold for the phasic model [36], [37]. Rather, the firing threshold is dynamic and involves *V*_m_ and *w* together, as affected by the input current. The full model (Equation 1) is multi-dimensional; however, by considering a reduced two-dimensional model [38], we reveal the dynamic threshold geometrically, as a separatrix curve in the *V*_m_-*w* plane. For this reduction, we suppose that the sodium current (*I*_Na_) activates instantaneously, i.e. we set *m* to 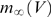. The nullclines and separatrixes are dynamic and move in this two-variable projection, depending on the stimulus and other dynamic variables. In order to demonstrate the dynamic aspects, we consider, first, the rising phase case and choose two points on the STA time course and trajectory (gray in Fig. 6 A–C, D, *right*): one slightly before (red circle) and one slightly after (purple circle) the initiation of a spike. The corresponding phase points in the signal's trajectory are also marked (Fig. 6D, *left*, triangles). In Fig. 6D, the nullclines and separatrixes were constructed with the variables *h*, *n*, *p*, *z*, and *r* set to their individual instantaneous values at the times chosen for the two “snapshots” (the circles/squares). For the STA-driven case, these values were obtained from trial-averaging of the respective variables over the spike-generating trajectories.

In the noise-free case, the threshold separatrix driven by signal alone moved upward as the signal increased (Fig. 6D, *left*, red to purple). However, the phase point for the signal alone (triangles) also moved upward and ahead of the separatrix; no threshold-crossing occurred and the system remained subthreshold. In contrast, the mean spike-triggering noise, first hyperpolarizing, pushed the trajectory (Fig. 6D, *right*, gray) toward the *w*-nullcline (blue solid). This push and proximity to the *w*-nullcline slowed the motion along the trajectory (i.e., d*w*/dt is small close to the nullcline), accounting for the slowed rise of the *V*_m_ and *w* time courses; while in the noise-free case (Fig. 6D, *left*) the trajectory was not slowed or close to the *w* nullcline. With this slowed growth of the *I*_KLT_ the phasic model was hyperexcitable compared to that in the noise-free case for the same signal values. When the STA noise became depolarizing, the separatrix moved upwards rapidly (Fig. 6D, *right*, red to purple), sweeping through the slowed phase point, thereby creating a threshold crossing.

The geometrical analysis for the threshold and response dynamics during the signal's falling phase is analogous, showing how the STA-noise accelerated the trajectory to enable spike generation. Just before a spike, the hyperpolarizing noise pushed the STA phase point down and leftward to become farther away from the *w* nullcline (Fig. 6E, *right*, squares) than in the noise-free case (Fig. 6E, *left*, triangles). This increased distance indicates that d*w*/dt was more negative in the STA-case, hence speeding up the motion and the decrease of *I*_KLT_. This accelerated decrease leads to a timely window for depolarizing fluctuations that, on average, swept the separatrix upwards through the phase point, creating a threshold crossing and spike.

Movies that show the dynamic phase planes (involving separatrixes, nullclines, and *V*_m_-*w* phase points) are included in the supplemental materials (Video S1). Although *I*_KLT_ played a major role in creating the above behavior, the inactivation of *I*_Na_, denoted by *h*, also made a small contribution in a way similar to *w*. For example, the hyperpolarizing noise slowed down the decrease of *h* in the rising phase and speeded up the increase of *h* in the falling phase. The phase-plane analysis in the *V*_m_-*h* plane is also included in the supplemental materials (Video S2).

### Input-Output Signatures of Slope-Based Stochastic Resonance

The above simulations showed that noise can enable the phasic model to encode low-frequency signals, which alone cause no response, in a way essentially different from the classical SR. In fact, the slope-based SR behavior might be inferred from other properties of the phasic model, such as the *f*-*I*, *f*-*A* (signal amplitude) and *f*-slope curves obtained in the presence of noise. These properties are usually studied in noise-free conditions; however, in reality more or less noise is always present for a neuron. Below we will compare these properties for the tonic and the phasic models in noisy conditions, and explain why they are related to the behavior of SR.

#### *a) f*-*I* curves

**Figure 7:**
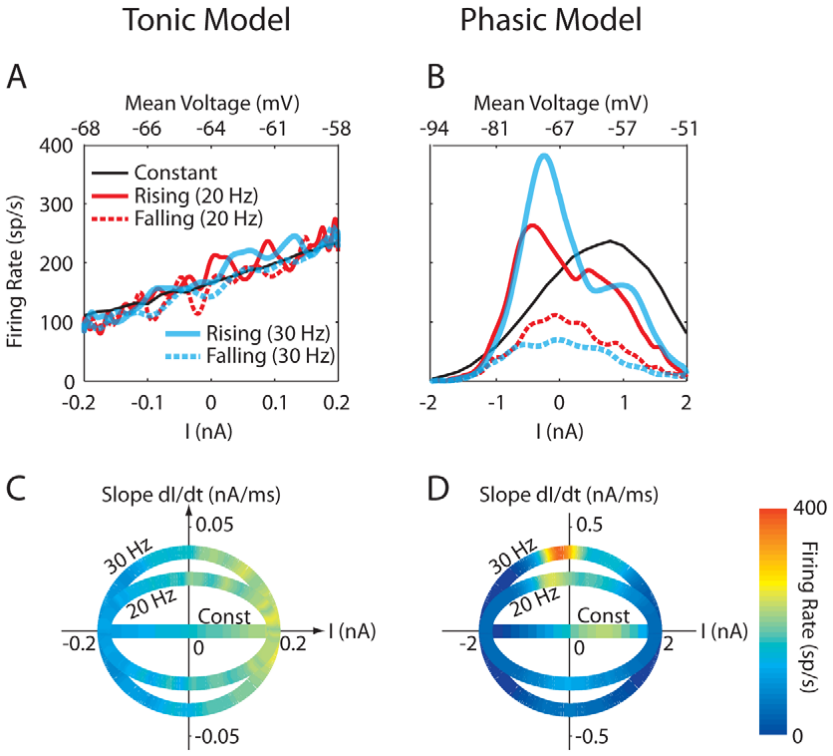
Firing rate as a function of input mean or input slope. (A) and (B) Firing rates vs. input mean (*I*, lower horizontal axis) in the presence of noise. Colored lines, instantaneous firing rate converted from period histograms when the input was white noise plus a 20 or 30 Hz sinusoid (*A* = 0.2 and 2 nA for the tonic and phasic models, respectively), which provided a noisy input with time-varying *I*. Spikes were separated for the signal's rising (solid) and falling (dotted) phases. Each phase covered a half cycle of the sinusoid. The curves were smoothed with Gaussian functions with a standard deviation of 0.5 ms. Black solid lines, average firing rate when *I* was fixed for 10 s. The top horizontal axis indicates the average *V*_m_ for fixed *I* values when the spiking mechanism was disabled by setting the activation/inactivation variables of the *I*_Na_ and *I*_KHT_ to their resting values. (C) and (D) Firing rates vs. *I* and slope (dI/dt) in the presence of noise. The firing rate is represented by the color. Noise *σ* was 10 and 15 pA for the tonic (left) and phasic (right) models, respectively.

Because phasic neurons do not show sustained responses to steady input current, an *f*-*I* curve cannot be obtained in a noise-free condition. However, *f*-*I* curves, which turn out to be highly non-monotonic, can be obtained with the phasic model when noise is added [24], [26], [27]. A typical *f*-*I* curve for white noise *σ* = 15 pA is plotted in Fig. 7B (black solid). Here *I* was the input mean, which was kept constant over 10 s during simulations. The maximum firing rate of the phasic model was reached at some intermediate *I* value (∼0.8 nA) rather than at the highest *I* value, in contrast to the *f*-*I* curve of the tonic model which increased monotonically with *I* (Fig. 7A). We believe that such a highly non-monotonic *f*-*I* curve exhibited by the phasic model (Fig. 7B) correlates with slope-based SR responses, because the monotonic curve exhibited by the tonic model (Fig. 7A) will inevitably lead to amplitude-based responses. We will test this hypothesis with other types of phasic models (see the last section of the Results).

According to the period histograms of the phasic model in responses to a 20-Hz signal with noise (Fig. 4, *right*, 2^nd^ column), temporal variation of *I* can affect the *f*-*I* curve, even when the variation is relatively slow (e.g., 20 Hz); otherwise, responses to the signal's rising and falling phases would be equal. Fig. 7B plots the *f*-*I* curve separated for spikes occurring at the signal's rising (solid) and falling phases (dotted) for the same noise *σ* (15 pA) for a 20- (red) or a 30-Hz (blue) signal. Differences can be observed between the *f*-*I* curves for constant *I* (black) and time-varying *I* (red or blue). Some of the differences were caused by different degree of the activation of slow cation current (*I*_h_) or the inactivation of the *I*_KLT_ comparing constant vs. time-varying *I*; others were caused by fundamental properties of the phasic model. We will separate these two factors below.

*Differences caused by I_h_ and the inactivation of I_KLT_*: When *I* was constant (Fig. 7B, black), the peak firing rate occurred at a positive *I* value (∼0.8 nA) and the phasic model showed some amount of spiking activity to the highest *I* value (i.e., 2 nA). The upper horizontal axis shows the average *V*_m_ for each *I* value when the spiking mechanism was disabled. The peak of the steady *f*-*I* curve corresponds to a *V*_m_ that was approximately 4 mV above the resting *V*_m_ (−64 mV). When *I* was periodic (red and blue), the model fired less to depolarizing *I* values and the peak firing rates occurred at negative *I* values (−0.4 to −0.1 nA). The STA of *V*_m_ shows that spikes were initiated when the *V*_m_ was approximately −70 mV. This difference was mostly caused by a higher input resistance with steady *I* due to deactivation of *I*_h_ when the *V*_m_ was continuously depolarized with *I*>0 (e.g., activation variable *r* 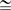
0.02 for *I* = 2 nA). When *I* was varying at 20 or 30 Hz, *I*_h_ was too slow to deactivate for positive *I* values. In addition, *z*, the slow inactivation of *I*_KLT_, for constant positive input (black) leads to easier spiking because of the smaller *I*_KLT_. Additional simulation results showed that when *z* and *r* were fixed, the peak firing rates for constant and for time-varying *I* occurred at the same *I* value (not shown).

*Differences caused by fundamental properties of the phasic model*: First, when the periodic *I* increased from hyperpolarizing to depolarizing values during the rising phase, more spikes occurred (Fig. 7B, red or blue solid) than the responses for constant *I* (black), because a previous hyperpolarization reduced the amount of *I*_KLT_. When the periodic *I* swept from depolarizing to hyperpolarizing values during the falling phase, the firing rate was always lower (red or blue dotted) than the responses for constant *I* (black) due to a higher amount of recruited *I*_KLT_. Second, the 30-Hz signal elicited a higher peak firing rate (blue solid) compared to the 20-Hz signal (red solid) for the signal's rising phase, indicating that the phasic model was indeed sensitive to the rising slope of the input mean. This result is better illustrated when dI/dt is also included as a parameter (Fig. 7D). The peak firing rate of the phasic model (indicated by the warm color) increased with dI/dt (y-axis). In contrast, the firing rate of the tonic model was not sensitive to dI/dt (Fig. 7C). The insensitivity of the tonic model to input slope explains why all the *f*-*I* curves obtained with different dynamics of the input mean lined up with each other (Fig. 7A).

#### *b) f*-*A* curves

As described above, the phasic model shows a non-monotonic *f*-*I* curve, either for steady *I* or for slowly varying *I*. The peak of the *f*-*I* curve may vary with the dynamic of *I*, but it is generally true that intermediate *I* values yield higher firing rates than low or high *I* values. If a time-varying signal, such as a 20-Hz sinusoid, has a mean that is close to the peak of the *f*-*I* curve, it can be predicted that increasing the amplitude of the signal will lead to decreased firing rate. This trend was observed with the phasic model when comparing the responses to noise alone, 1-nA, and 2-nA signals (Fig. 3A, *right*). Here we further study this issue by creating the *f*-*A* curves, where *A* is the peak amplitude of the 20-Hz sinusoid. In the Discussion, we will explore the practical meanings of these curves in terms of the linearization effect of noise and the maximization of input/output dynamic range.

**Figure 8:**
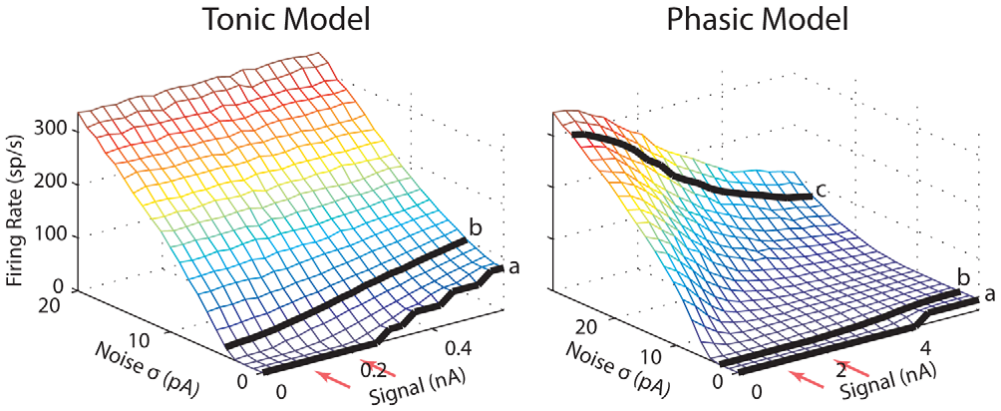
Average firing rate vs. signal amplitude for different noise intensity (*σ*). The signal is a 20-Hz sinusoid. The two orange arrows indicate the signal amplitudes used in the previous simulations for the noise-gated signal encoding. Duration of the stimulus was 10 s.

Fig. 8 shows the *f*-*A* curves obtained with different amount of noise. The tonic model had increasing *f*-*A* curves for low-level noise (e.g., black lines marked with ***a*** and ***b***) and almost constant *f*-*A* curves for high-intensity noise (Fig. 8, *left*). The near constant *f*-*A* curves for strong noise was due to the almost linear *f*-*I* curves (Fig. 7A). When *I* varies in a larger range surrounding 0 nA, the increase of firing rate with positive *I* values (e.g., at the signal's peak) was approximately the same as the decrease of firing rate with negative *I* values (e.g., at the signal's trough). In contrast, the phasic model showed increasing *f*-*A* curves only for weak noise, and the change of the firing rate was small (Fig. 8, *right*, black lines marked with ***a*** and ***b***), because the phasic model fires one spike in each signal cycle for most of its input conditions (Fig. 1B, *right*). It typically did not fire to a 20-Hz signal unless the signal amplitude was extremely large (>4 nA), and even in this case the firing rate only reached 20 sp/s (Fig. 8, *right*, ***a***). At medium and high noise intensities, the *f*-*A* curves became monotonically decreasing, and the decrease was large (***c***).

#### *c) f*-slope curves for ramp stimuli

**Figure 9:**
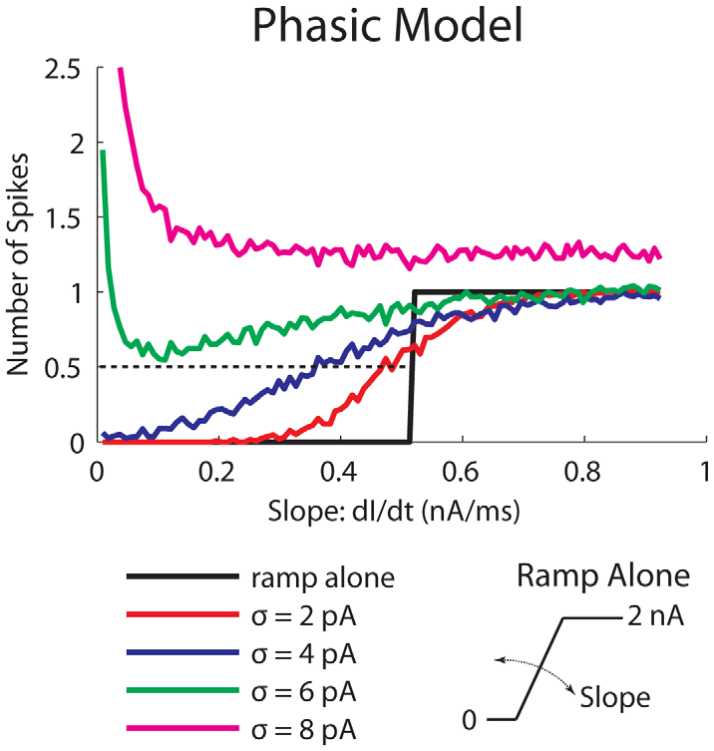
Number of spikes vs. slope of the ramp stimulus for the phasic model when white noise of different intensity (σ) was added to the ramp. Intersections between the solid lines and the black dotted line define the slope threshold. The small plot on the lower right shows the ramp stimulus without noise. Number of spikes was measured during the sloping part of the stimulus and was averaged over 200 repetitions. Note that the duration of the sloping part for spike counting varies with the slope. The large number of spikes when strong noise was added to a ramp with shallow slopes was caused by responses to the noise within a long spike-counting window.

In the above simulations we used sinusoidal signals. We believed that the slope-based SR can also be observed with other types of time-varying signals that have only low-frequency components. As shown in Fig. 2, the phasic model does not respond to ramp stimuli with slopes shallower than 0.55 nA/ms. In other words, there is an input threshold in terms of slope that yields a step-like *f*-dI/dt curve (Fig. 9, black solid). With noise added, the *f*-dI/dt curves were smoothed (Fig. 9, colored lines). For weak noise (*σ*≤6 nA), the firing rate increased with input slope, similar to what was observed with periodic signals (Fig. 7D).

Here we computed the average number of spikes (200 repetitions) in response to a rising ramp with white noise added. Since the slopes around the threshold (0.55 nA/ms) were steep, no more than one spike can occur. We arbitrarily picked up a criterion of 0.5 number of spikes (for 50% of the trials there was a spike; Fig. 9, dotted), for defining the slope threshold. With increasing amount of noise the slope threshold decreased and eventually disappeared for *σ*≥6 pA (Fig. 9).

### Generalization of Findings to Other Phasic Models

The phasic model used in the above simulations derives its Class 3 excitability through a negative feedback current, the *I*_KLT_, which activates below spike threshold. Here, we tested whether another two types of phasic models show similar slope-based SR behavior.

First, we tested a phasic HH model with a steeper activation of the *I*_K_ [25] compared to the original HH model [39]. The modification of *I*_K_ is to achieve the Class 3 excitability, which was observed with squid giant axons but not with the original HH model. With a simulation temperature of 18.5°C, the phasic HH model showed a slope threshold around dI/dt = 3.5 (mA/ms)/cm^2^ for ramp inputs increasing from 0 to 50 mA/cm^2^. When noise of different intensities was added, the slope threshold decreased and further disappeared (for noise *σ*≥50 nA/cm^2^) in a way similar to the behavior of the phasic model (Fig. 9), except that multiple spikes occurred during the ramp (not shown).

**Figure 10:**
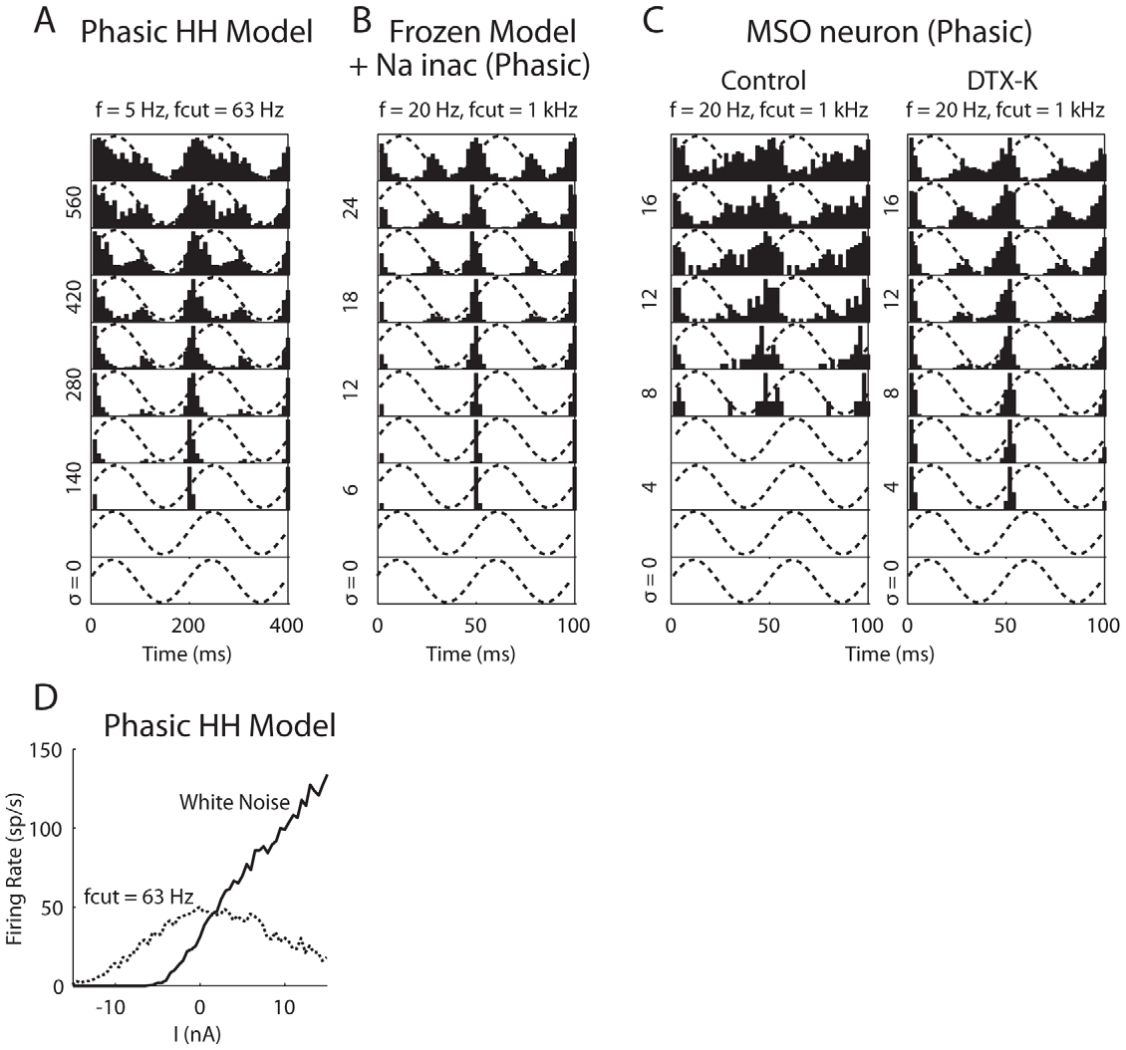
Responses of other models and neuron to a subthreshold signal with noise. (A–C) Period histograms in response to subthreshold signals with different amount of noise added. (A) The phasic Hodgkin and Huxley (HH) model (Clay et al. 2008) at 18.5°C. The signal was a sinusoid with *A*_s_ = 15 nA/cm^2^. (B) A new phasic model created from the tonic model (*I*_KLT_ was frozen) by shifting the voltage dependency of the sodium inactivation by −15 mV at 32°C. The signal was a sinusoid with *A*_s_ = 2 nA. (C) An MSO neuron recorded in a brain slice from a gerbil aged P18 before and after 60 nM DTX-K was bath applied at 32°C. The signal was a modified sinusoid (the negative part of the sinusoid was multiplied by a factor of 0.5) with *A*_s_ = 1.5 nA. The dotted lines are superimposed signals scaled to illustrate the response phase. (D) f-I curves of the phasic HH model obtained with white noise (*σ* = 100 pA/cm^2^) and low-pass filtered noise (*σ* = 500 pA/cm^2^). *f*, signal frequency. *f*_cut_, cutoff frequency of the low-pass filtered noise. Noise *σ* [in pA/cm^2^ in (A) and pA in (B) and (C)] is measured with the white noise before low-pass filtering.

The phasic HH model also showed a band-pass filtering property for noise-free sinusoidal input (not shown) similar to that of the phasic auditory brainstem model (Fig. 1C, *right*), except that multiple spikes can occur in each signal cycle at medium-low frequencies. The phasic HH model did not fire to a 5-Hz sinusoidal signal up to 28 mA/cm^2^ and the voltage trace showed similar rectification as exhibited by the auditory brainstem model (Fig. 1B, *right*). When white noise was added to a subthreshold signal (*A*_s_ = 15 mA/cm^2^), the negative feedback created by the *I*_K_ was not strong or fast enough to prevent spikes at the signal's peak (not shown). However, after the white noise was low-pass filtered with a cutoff frequency of <100 Hz, the phasic HH model also showed highest sensitivity to the signal's rising and falling phases and low response to the peak (Fig. 10A), which resembled the temporal pattern obtained from the phasic auditory brainstem model (Fig. 4, *right*). Correspondingly, the *f*-*I* curve of the phasic HH model was monotonically increasing with white noise (Fig. 10D, solid) but started becoming non-monotonic when *f*_cut_ was lowered to 100–150 Hz. Fig. 10D (dotted) shows an example non-monotonic *f*-*I* curve with the peak at 0 and minimal firing at ±15 mA/cm^2^ (*f*_cut_ = 63 Hz). The change of the *f*-*I* curve with noise spectrum confirmed that a non-monotonic *f*-*I* curve correlates with the slope-based SR for a certain noise profile.

It should be pointed out that in previous physiological and computational studies showing the non-monotonic *f*-*I* curves [24], [26], [27], Gaussian white noise was smoothed by exponential filters with *τ* = 1–3 ms. The spectrum of noise created this way is a decreasing function of frequency. Repeating the phasic HH model with smoothed white noise showed non-monotonic *f*-*I* curves for *τ* = 1–3 ms.

A third cellular mechanism that can create Class 3 excitability is the inactivation of *I*_Na_ [23], [24]. To test the role of *I*_Na_ inactivation alone in creating phasicness and the slope-based SR behavior, we shifted the sodium inactivation voltage sensitivity (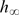) leftward by 15 mV [23], while freezing the conductance of the *I*_KLT_ to its resting value as we did for the tonic model. These manipulations created the Class 3 excitability and the slope-detecting ability (a slope threshold around dI/dt = 0.22 nA/ms for ramp inputs increasing from 0 to 2 nA in a noise-free condition). When white noise was added to a 20-Hz subthreshold signal (*A*_s_ = 2 nA), the model fired most at the signal's rising and falling phases, with less activity at the peak (not shown). Lowering the cutoff frequency of the noise decreased the activity at the peak (an example plotted in Fig. 10B for *f*_cut_ = 1 kHz). Correspondingly, the *f*-*I* curve with white noise was increasing for most *I* values and decreased slightly for I>1.5 nA, while the *f*-*I* curve with low-pass filtered noise was highly non-monotonic (not shown). In addition, a previous computational study [24] showed that by lowering the conductance of *I*_Na_ from 120 to 83 mS/cm^2^, the standard HH model can also exhibit Class 3 excitability and non-monotonic *f*-*I* curves for exponentially smoothed noise (*τ* = 1 ms). We simulated this model with low-pass filtered noise and found behaviors similar to what was described above for the phasic HH model with modified *I*_K_.

Principal neurons in the MSO are shown to have both low-voltage inactivation of *I*_Na_ [23] and *I*_KLT_. With blockade of the *I*_KLT_, the inactivation of *I*_Na_ alone can cause MSO neurons to show phasic response for gerbils of postnatal day (P) 14 or 15 and older, but becomes more prominent for neurons >P17 [23]. In our previous study [14], we showed that *I*_KLT_ plays a major role in creating the slope-based SR response for MSO neurons of P14–16. Here we repeated the experiments with three neurons from older animals (P18–20), for which *I*_Na_ is known to be highly inactivated. Fig. 10C (*left*) shows that in response to a 20-Hz signal (*A*_s_ = 1.5 nA), the neuron (P18) fired mostly to the signal's rising, falling phases and the trough. Low-pass filtered noise (*f*_cut_ = 1 kHz), instead of white noise, was used in the recordings because the electrode was not fast enough to generate white noise [14]. The neuron fired in the signal's trough because its membrane time constant (0.3 ms) was so fast that it can integrate slow noise fluctuations even when the neuron was somehow hyperpolarized [14]. After the application of dendrotoxin-K (DTX-K, a blocking agent selective for *I*_KLT_), the neuron started responding to lower noise intensities at the signal's rising phase (Fig. 10C, *right*) and clear firing preferences to the rising and falling phases can be seen for all noise intensities. Less firing in the trough was observed due to a steeper *V*-*I* relationship after DTX-K was applied [23]. For example, in the control condition at the signal's minimum the neuron was hyperpolarized by −5 mV from the resting potential, while the hyperpolarization increased to −15 mV by the same signal after DTX-K was applied, too far from spike threshold for noise to elicit spikes in the trough. Note that in the recordings the signal's negative part was scaled by a factor of 0.5 to prevent large hyperpolarizations. Similar results were obtained in the other two neurons recorded.

## Discussion

Phasic neurons do not respond to constant or slowly varying inputs in the absence of noise [10]–[12]. Recently we showed experimental and computational evidence that noise enables phasic neurons to encode low-frequency signals [14]. Here we introduce the notion of a slope-based stochastic-resonance (SR) for low-frequency signals and characterize it with three types of phasic neuron models [15], [23]–[25]. Our work extends the classical, amplitude-based, SR theory to include noise-gated responses of phasic (i.e., Class 3) neurons.

### Slope-Based SR with Phasic Neurons vs. Amplitude-Based SR with Tonic Neurons

Tonic neurons show classical SR behavior; that is, noise helps the detection of a subthreshold signal by generating spikes when the signal is near its peak, i.e., when the *V*_m_ is closest to the firing threshold. This type of noise-controlled signal encoding is qualitatively similar to enlarging the amplitude of the signal. Indeed, the amplitude-frequency plot for sinusoidal input (Fig. 1C, *left*) shows a relatively constant input threshold (∼0.3 nA) except at the high-frequency end. Thus enlarging the signal amplitude sufficiently can make the model respond to the signal even in the absence of noise. In contrast, such an input threshold in terms of input amplitude does not exist for the phasic model (Fig. 1C, *right*); for sufficiently low-frequency signals, enlarging the signal amplitude does not make the model fire. Consequently, classical SR theory does not capture the signal response of noisy phasic models.

It is more appropriate to define the input threshold for a phasic model in terms of input slope or frequency. Adding noise to a signal with a frequency/slope below this threshold makes the phasic model fire, not because adding noise effectively enlarges the signal amplitude, but because noise transiently increases the slope/speed of the signal, or equivalently diminishes the slope/frequency threshold (Fig. 9). In this sense, it is not surprising to see that the phasic model is most sensitive to the signal's rising phase, where the slope of the signal is steep, rather than to the signal's peak, where the slope is zero (Fig. 4, *right*).

Based on our findings, we call the noise-gated encoding of a low-frequency signal by phasic models a “slope-based SR”, in contrast to the general (amplitude-based) SR observed in tonic models. Classical SR theory for weak and slow signals fails to explain both the SNR curve and the temporal firing patterns for the phasic model. This failure is because the SNR, computed either at the signal's fundamental frequency or harmonics, does not capture the full firing properties of the phasic model.

### A Non-Monotonic *f*-*I* Curve Correlates with Slope-Based SR

Previous studies [24], [26], [27] have shown that, in the presence of noise, tonic-firing neurons have monotonically increasing *f*-*I* curves, while phasic-firing neurons (i.e., Class 3) have highly non-monotonic *f*-*I* curves (Fig. 7). Because a monotonically increasing *f*-*I* curve predicts that the neuron will respond mostly to the peak of a low-frequency signal, we hypothesize that a non-monotonic *f*-*I* curve with peak firing rate at moderate *I* is suggestive of a slope-based SR. This hypothesis is supported by the phasic HH model and the phasic model with low-voltage inactivation of *I*_Na_, for which by varying the cutoff frequency of the low-pass filtered noise, the monotonicity of the *f*-*I* curve is correlated to the slope-based SR behavior (Fig. 10).

Some predictions for the phasic models can be derived from the *f*-*I* curves obtained with steady and time-varying *I*. First, in the quasi-static limit, the amount of firing in the falling phase should be equal to the firing in the rising phase. In contrast, with increasing signal frequency, firing in the rising phase should increase, while firing in the falling phase should decrease and eventually disappear. This trend was shown by the phasic model with the *I*_KLT_ in response to the 20- vs. the 30-Hz signals (Fig. 7). Second, when the signal amplitude was small (i.e., a smaller range of *I*), the slope-based SR behavior will be less distinct compared to the response with large signal amplitude. Consistent with this prediction, we showed in our previous study [14] that the responses of the phasic auditory brainstem model to the signal's rising and falling phases were less distinct when the signal amplitude decreased from 2 to 1 nA.

Previous studies describing non-monotonic *f*-*I* curves have not addressed the influence of the noise spectrum on firing rate [24], [26], [27]. In general, the fact that phasic models can remain sensitive to the slope of subthreshold (low-frequency) signals in the presence of noise is a continuity of phasicness as defined in a deterministic setting. Although all the phasic models tested here showed Class 3 excitability, we propose different degrees of “phasicness” characterized by a model's resistance of losing the non-monotonicity of the *f*-*I* curves when the noisy fluctuations become faster. For example, the phasic model with *I*_KLT_ has the strongest phasicness among all phasic models studied because it maintained its phasicness (detecting slopes and onsets) even if the noise fluctuated as fast as a 25-kHz white noise. The other phasic models can maintain their phasicness only when the noise was relatively slow. However, low-pass filtered noise is a better model of neural fluctuations than white noise, because noisy input to neurons, in the form of random background synaptic events, is naturally spectrally limited due to synaptic filtering [40]. Therefore, the slope-based SR may be widely present with phasic neurons, and that a highly non-monotonic *f*-*I* relation can serve as an indicator. Note that here the degree of phasicness is different from the definitions used in other studies [29], [31]. In those studies the term is used to describe the firing properties of a phasic neuron to noise-free step input when multiple spikes can occur at the onset, whereas in our study all the phasic models fired only one spike at the onset.

### Phasic Neurons as Slope Detectors

Phasic neurons are labeled slope-detectors based on their sensitivity to the slope of a ramp current. That is, they do not respond to a current input that has a slope shallower than a threshold value even if the input amplitude is large [12], [28]. Although this concept was developed for auditory brainstem neurons [28], it is a characteristic of all phasic neurons, because if a steady current input causes no response, by continuity there will be no spiking for sufficiently slow ramp input. We showed that the slope threshold is lowered in the presence of weak noise, and further diminished when noise is strong enough to cause significant spiking in the absence of a signal (Fig. 9). When the slope threshold is lowered, a phasic neuron can respond to inputs that are below the threshold obtained without noise. In a classical amplitude-based SR system, noise brings the system above its amplitude threshold, effectively mimicking an *upward* shift in the response-area plot in Fig. 1C (*left*). Correspondingly, in a slope-based SR system, noise brings the system above its slope threshold, which effectively moves in the *rightward* direction in the response-area plot in Fig. 1C (*right*).

The sensitivity of phasic models to input slopes can also be clearly observed in the *f*-*I*-dI/dt plots obtained with time-varying *I* (Fig. 7D). The peak firing rate of the phasic models increased with dI/dt, while the firing rate of the tonic model was insensitive to dI/dt. The higher firing rate on the rising phase of the 30-Hz signal compared to the firing rate to the 20-Hz signal indicated that the 30-Hz signal was closer to the input threshold in terms of the frequency of a sinusoid (i.e., 32 Hz for *A*_s_ = 2).

### Noise Enlarges Input/Output Dynamic Range Differently for Tonic and Phasic Models

Based on the non-monotonic noise-based *f*-*I* curves obtained with the phasic auditory brainstem model (Fig. 7B), we predicted that the firing rate will decrease with increasing amplitude of a sinusoidal signal for moderate and strong noise (Fig. 8, *right*). In contrast, the tonic model showed increasing *f*-*A* curves at low noise intensities and relatively constant curves at high noise intensities (Fig. 8, *left*). These differences in the *f*-*A* curves indicate that noise plays a different role in affecting input-output relationships for tonic vs. phasic neurons.

When firing rate encodes the amplitude of a periodic input (*A*), the input dynamic range that evokes spikes is limited, since the firing rate remained zero when *A* varies below threshold (Fig. 8, black lines marked with ***a***). Adding noise to the input linearizes the *f*-*A* curve, thereby achieving a larger input dynamic range [1], [41]–[43]. For the tonic model, adding a small amount of noise (e.g., 4 pA) can achieve this type of linearization, so that the firing rate increases with a relatively constant rate even in the subthreshold regime (Fig. 8, *left*, ***b***). When the noise intensity further increases, the output dynamic range decreases as the firing rate becomes insensitive to *A*.

For the phasic model, although a similar trend exists for weak noise (e.g., *σ* = 3 pA), the input dynamic range increased only around the input threshold (4 nA). The output dynamic range was also limited because the maximum firing rate was close to the signal frequency even with a small amount of noise added (Fig. 8, *right*, ***b***). However, with strong noise added, the firing rate decreased relatively smoothly with *A* (Fig. 8, *right*, ***c***) and a large range of *A* can be encoded by the firing rate. In addition, because a large amount of noise can cause multiple spikes in each signal cycle, a large output dynamic range was also achieved (Fig. 8, *right*, ***c***).

It should be noted that although the firing rate decreases, the temporal precision increases with *A*. The two groups of spikes on the signal's rising and falling phases were less distinct for *A_s_* = 1 nA compared to the 2-nA case [14], although the SNRs were comparable (Fig. 3B, *right*). The maximum slope of the input increases when signal frequency is kept constant and the signal amplitude is enlarged; however, the fraction of time spent around the maximum slope decreases, leading to a narrower time-window for the first crossing of the slope threshold.

### Significance of Slope-Based SR in Nervous System

Phasic neurons are widely present in different sensory systems (for review, see [29]). These neurons are thought to detect onset events, encode fast changes of its input, and maintain response temporal precision [28], [29]. Our results suggest that in the presence of noise, phasic neurons can encode slow inputs and remain sensitive to changes of an input (e.g., the beginning and end of the positive cycle of a sinusoid), thereby extending their phasicness into the low-frequency region. The slope-based SR behavior reflects the tendency of phasic neurons to maintain its phasicness when moderate noisy fluctuations are applied; large intensity noise will, of course, reduce slope detection. In summary, slope-based SR is a continuity of phasic neurons' response properties over large input dynamics.

Noise-gated or noise-assisted coding, of which SR is a classic example, is a popular topic in neural applications and other physical systems, where simple threshold models serve as a canonical model for SR [4], [44]. Phasic systems offer a new avenue of research in noise-assisted coding, where the distinction of signal threshold is based on the slope of the signal, rather than the more traditional scenario of an amplitude threshold. We expect that new noise induced phenomena, qualitatively distinct from those previously described, will emerge as noise-driven phasic systems are studied further.

## Methods

### Neuron Models

Details of the phasic neuron model are described in [14]. Briefly, the auditory brainstem neuron model [15] contains a fast sodium current (*I*_Na_), a high-threshold (*I*_KHT_) and a low-threshold (*I*_KLT_) potassium currents, a hyperpolarization-activated cation current (*I*_h_), and a leak current (*I*_lk_).
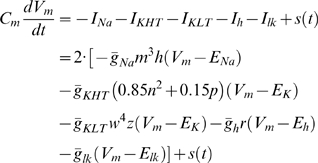
(1)


*V*_m_ is the membrane voltage. 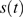 is the current input. Membrane capacitance, 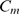 = 12 pF; maximal channel conductances, 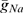 = 1000 nS, 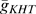 = 150 nS, 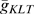 = 200 nS, 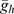 = 20 nS, and 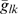 = 2 nS; reversal potentials, 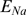 = +55 mV, 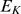 = −70 mV, 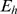 = −43 mV, and 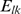 = −65 mV. All the conductances and channel time constants are multiplied by a factor of 2 and 0.33, respectively, to mimic the condition at 32°C, because our previous study [14] had slice recordings at 32°C. Although there are several currents with time-varying conductances, only the fast component of the *I*_KLT_, *w*, was playing a major role in the simulations with sinusoidal and noisy inputs [14].

The tonic model is created by fixing the gating variables, *w* and *z*, to the values obtained at resting potential [45]. A further modification of the Day's frozen model is to increase the 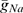 from 1000 to 1500 nS, which enables a larger amplitude of the limit cycle and a broader input range for repetitive firing [14]. The tonic model created this way has the same membrane resting potential and input resistance as the phasic model does.

### Input Stimulus for Noise-Gated Encoding of Signal

The signal was a 20-Hz sinusoidal current (unless otherwise specified), 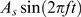, with zero mean. The signal was kept subthreshold, and white noise (0–25 kHz) was added to make the model spike.
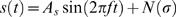
(2)Note that in our previous study [14] and the present physiological recordings, the negative part of the signal is multiplied by a factor of 0.5 to avoid excessive hyperpolarization of the neuron in whole-cell recordings. Here we used the unmodified sinusoid in the simulations because we were trying to make a direct comparison with classical SR systems where pure sinusoidal inputs are commonly used as signals.

**Figure 11:**
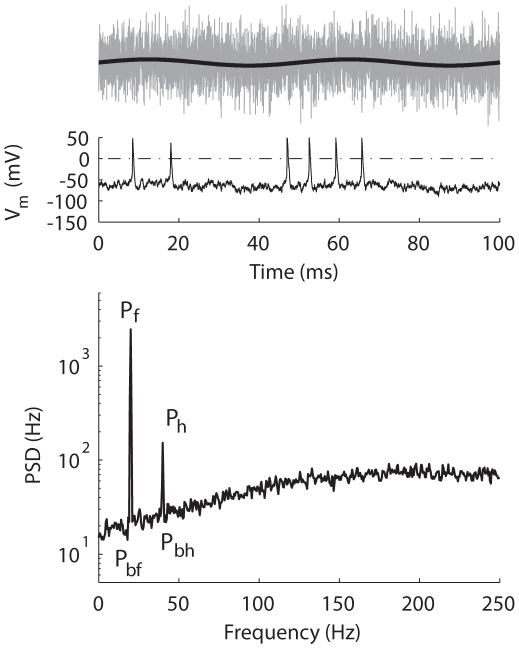
An example of power-spectrum density (PSD). *Top*, the signal (black) and the signal plus noise (gray). *Middle*, *V*_m_ (solid) and the voltage level that identified a spike (dotted). *Bottom*, PSD for the tonic model in response to a 20-Hz signal (*A* = 0.2 nA) with white noise (σ = 5 pA). *P_f_*, peak of the fundamental. *P_h_*, peak of the first harmonic. *P_bf_*, baseline for the fundamental. *P_bh_*, baseline for the harmonic. Frequency resolution = 0.5 Hz. Total duration = 100 s. Only the first 100 ms of stimulus and response are shown in *top* and *middle* panels.

Two signal amplitudes were chosen (*A_s_* = 0.1 and 0.2 nA for the tonic model and *A_s_* = 1 and 2 nA for the phasic model) so that the detectability of signal from noise (quantified by the signal-to-noise ratio) was comparable between the two models. Our choice of the input amplitude for the phasic model is reasonable because MSO neurons, the phasic neurons that demonstrated similar properties compared to the phasic model [14], have an average input threshold of 3–4 nA for step input [46]. The sampling frequency was 50 kHz. For each noise intensity, ∼5000 spikes were obtained unless stimulus duration reached 200 s. Fig. 11 shows an example of input stimulus (*top*) and the corresponding response of the tonic model (*middle*).

### Signal-to-Noise Ratio (SNR)

The detectability of the signal from the added noise was quantified by computing the signal-to-noise ratio (SNR) from the power spectrum of the spike train. Fig. 11 (*bottom*) shows an example of power-spectrum density (PSD) computed from the spike times of the tonic model. Spike times were re-sampled with a lower time resolution, 2 ms, producing a Nyquist of 250 Hz in the PSD plot (higher frequency was unnecessary since the signal frequency was low). Two peaks are visible in the PSD plot, one at the signal frequency (marked as *P_f_*) and another at the first harmonic (marked as *P_h_*). The SNR is computed as
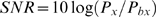
(3)where x is either the fundamental frequency (x = f), or the first harmonic frequency (x = h). *P_bf_* is the baseline for the fundamental, computed as the average of a small range near *P_f_*, and *P_bh_* is the baseline for the first harmonic, computed as the average of a small range near *P_h_*. In the following text, SNR will refer to the signal-to-noise ratio at the fundamental frequency unless otherwise specified.

For simple spiking systems an adiabatic theory (slow signal) with weak signal amplitude approximates SNR as [4]

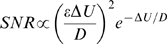
(4)where *ε* is the signal amplitude, *D* is the noise intensity (corresponding to the *σ^2^/2* in the present study), and *ΔU* is the potential barrier separating the deterministic rest state and firing threshold. When Equation 4 was used to fit a SNR curve, *D* is chosen so that the peak of Equation 4 overlaps the peak of the SNR. Specifically, because Equation 4 reaches its peak when *D* = *ΔU/2*, *ΔU* is chosen as twice the noise intensity where the peak of the SNR is obtained. Then the whole equation is scaled to match the peak SNR value. Note that Equation 4 predicts that the peak of the SNR is obtained with a fixed noise intensity invariant of the signal amplitude [32].

Another frequently used quantification of SR is the spectral power amplification (SPA) [9]. It is the output power at the signal frequency normalized by the input signal power,
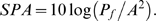
(5)Note that here the output power was the power of a discrete signal (i.e., spike times), while the input power was the power of a continuous signal (i.e., a sinusoidal current).

### Whole-Cell Recordings

*In vitro* data presented here is to confirm that strong sodium inactivation can replace *I*_KLT_ to create phasic responses. Detailed experimental procedures are described in [14]. Briefly, gerbils (*Meriones unguiculatus*) aged P17–18 were used to obtain 150-µm brainstem slices. The internal patch solution contained (in mM) 127.5 potassium gluconate, 0.6 EGTA, 10 HEPES, 2 MgCl_2_, 5 KCL, 2 ATP, 10 phosphocreatinine, and 0.3 GTP (pH 7.2). During recordings, sliceswere placed in a chamber with artificial cerebrospinal fluid (ACSF) containing (in mM) 125 NaCl, 4 KCl, 1.2 KH_2_PO_4_, 1.3 MgSO_4_, 26 NaHCO_3_, 15 glucose, 2.4 CaCl_2_, and 0.4 L-ascorbic acid (pH 7.3 when bubbled with 95% O_2_ and 5% CO_2_) at 32±1°C. DTX-K (60 nM) was added to the bath to block the *I*_KLT_. The perfusing rate of the oxygenated ACSF in the recording chamber was 2ml/min. An Axoclamp2A amplifier, in combination with Labview (National Instruments), was used for stimulus generation, balance of series resistance, and data acquisition at 10 kHz.

## Supporting Information

Video S1 Movie of *V*_m_-*w* phase plane. Top, STA of stimulus. Lower, STAs of *V*_m_-*w* phase planes for spikes occurring in a 4-ms window centering at the rising (gray) and falling (black) phases of the 20-Hz signal (*A*_s_ = 2 nA) for the phasic model. The signal alone and its responses are plotted in green. A representative phase point is marked with a circle (for rising phase) or a square (for falling phase). The corresponding phase point in the signal's trajectory is marked with triangle. V-null (blue solid), *V*_m_ nullcline. w-null (blue dotted), *w* nullcline. Red, threshold separatrix. The stimulus condition is as marked with *c* in Fig. 3. Stimulus duration was 500 s. Noise σ was 15 pA.
(2.53 MB WMV)

Video S2 Movie of *V*_m_-*h* phase plane. Top, STA of stimulus. Lower, STAs of *V*_m_-*h* phase planes for spikes occurring in a 4-ms window centering at the rising (gray) and falling (black) phases of the 20-Hz signal (*A*_s_ = 2 nA) for the phasic model. The signal alone and its responses are plotted in green. A representative phase point is marked with a circle (for rising phase) or a square (for falling phase). The corresponding phase point in the signal's trajectory is marked with triangle. V-null (blue solid), *V*_m_ nullcline. h-null (blue dotted), *h* nullcline. The stimulus condition is as marked with *c* in Fig. 3. Stimulus duration was 500 s. Noise σ was 15 pA.
(1.99 MB WMV)
